# Cytotoxic Activity of Ursolic Acid Derivatives Obtained by Isolation and Oxidative Derivatization

**DOI:** 10.3390/molecules18088929

**Published:** 2013-07-26

**Authors:** Kishor Mazumder, Katsunori Tanaka, Koichi Fukase

**Affiliations:** 1 Department of Pharmacy, University of Science and Technology Chittagong, Foy’s Lake, Chittagong 4202, Bangladesh; E-Mail: k.mazumder@pharmacy.ustc.ac.bd; 2 Department of Chemistry, Graduate School of Science, Osaka University, 1-1 Machikaneyama, Toyonaka, Osaka 560-0043, Japan; 3 RIKEN Advanced Science Institute, 2-1 Hirosawa, Wako-shi, Saitama 351-0198, Japan; E-Mail: kotzenori@riken.jp

**Keywords:** ursolic acid, *Saurauja roxburghii*, cytotoxicity, C6 rat glioma cell line, A431 human skin carcinoma cell line, dioxoruthenium(VI) tetraphenylporphyrin, biomimetic oxidation, cytochrome P450

## Abstract

Structure-activity relationships of ursane-type pentacyclic triterpenes obtained from natural sources and by chemical derivatization are reviewed. Ursolic acid, corosolic acid, and a new ursane-type pentacyclic triterpene, 7,24-dihydroxyursolic acid, were isolated from the methanolic extract of the leaves of the Bangladeshi medicinal plant, *Saurauja roxburghii*. Derivatization of ursolic acid by oxidation with dioxoruthenium (VI) tetraphenylporphyrins was investigated. Oxidation selectivity on the terpene structure was modulated by the auxiliaries introduced on the tetraphenylporphyrin. The natural triterpenes and oxidized derivatives were tested for cytotoxicity against the C6 rat glioma and A431 human skin carcinoma cell lines. Although they have the same ursane-type pentacyclic triterpene cores, the position and numbers of hydroxyls on the terpene structures significantly affected the activity and the selectivity towards the tested cell lines.

## 1. Introduction

Exploration of novel drugs or drug leads from Nature has been the major subject in natural product chemistry [1]. Some possible sources of natural products include plants, marine organisms, microbes and fungi. Of the approximately 250,000 higher species of plants it is estimated that only 5%–15% have been investigated for natural products. Only 20% of the marine organisms, which cover more than 70% of the Earth’s surface have been investigated [2]. Also, research suggests that less than 1% of bacterial species and less than 5% of fungal species are currently known [3]. Therefore, it is important that natural product chemistry continue to explore these natural resources in search of new natural products.

Plants have been utilized as medicines for thousands of years. These medicines initially took the form of crude drugs such as tinctures, teas, poultices, powders, and other herbal formulations [2,3]. The specific plants to be used and the methods of application for particular ailments were passed down through oral history. Eventually information regarding medicinal plants was recorded in herbals. In more recent history, the use of plants as medicines has involved the isolation of active compounds, beginning with the isolation of morphine from opium in the early 19th century [2,4]. Drug discovery from medicinal plants led to the isolation of early drugs such as cocaine, codeine, digitoxin, and quinine, in addition to morphine, of which some are still in use [2,5,6].

**Figure 1:**
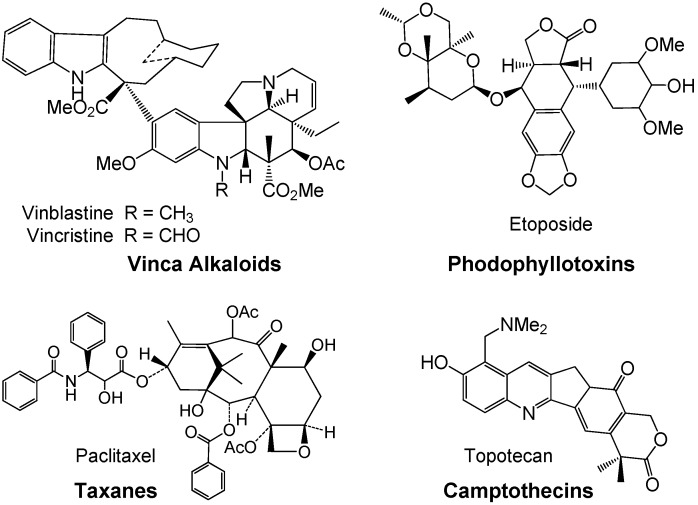
Plant derived anti-cancer agents: Four main classes of natural products.

Drug discovery from medicinal plants has played an especially important role in the treatment of cancer and, indeed, most new clinical applications of plant secondary metabolites and their derivatives over the last half century have been applied towards combating cancer. Of the all available anticancer drugs between 1940 and 2002, 40% were natural products or natural product-derived, with another 8% considered natural product mimics [5,6,7]. Anticancer agents from plants currently in clinical use can be categorized into four main classes of compounds: vinca (or Catharanthus) alkaloids, epipodophyllotoxins, taxanes, and camptothecins (Figure 1). Vinblastine and vincristine were isolated from *Catharanthus roseus (L.)* G. Don (Apocynaceae) (formerly *Vinca rosea* L.) and have been used clinically for over 40 years [8]. Podophyllotoxin was isolated from the resin of *Podophyllum peltatum* L. (Berberidaceae) but was found to be too toxic in mice so derivatives were made, with the first clinically approved drug being etoposide [9]. Paclitaxel was originally isolated from *Taxus brevifolia* Nutt. (Taxaceae) and was clinically introduced to the U.S. market in the early 1990s. Camptothecin was isolated from *Camptotheca acuminata* Decne. (Nyssaceae) but originally showed unacceptable myelosuppression [10,11,12]. Interest in camptothecin was revived when it was found to act by selective inhibition of topoisomerase I, involved in cleavage and reassembly of DNA [13]. Together, the taxanes and the camptothecins accounted for approximately one-third of the global anticancer market in 2002, over 2.75 billion dollars. Numerous derivatives of all four compound classes have been synthesized, some of which are currently in clinical use. All of these natural products have led to significant biological discoveries related to their unique mechanisms of action.

**Figure 2:**
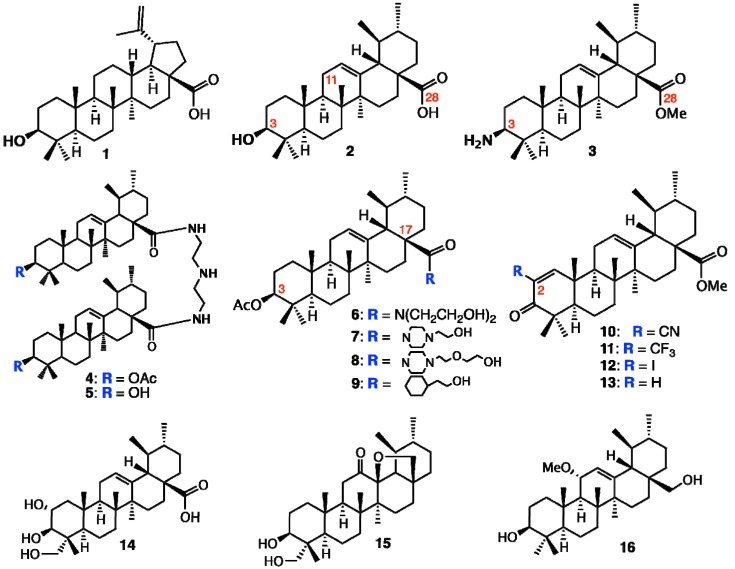
Structures of betulinic acid, ursane-type pentacyclic triterpenes, and derivatives.

Alternatively, the pentacyclic triterpenes are one group of promising secondary plant metabolites for cancer treatment. The triterpenes belonging to the lupane, oleanane or ursane groups have the potential to treat the cancer by different modes of action. Since Pisha* et al.* [14] reported in 1995 that betulinic acid (**1**) is a highly promising anticancer drug after inducing apoptosis in melanoma cell lines* in vitro* and* in vivo* (Figure 2), experimental work has focused on the apoptosis-inducing mechanisms of betulinic acid and other triterpenes. The antitumor effects were subsequently confirmed in a series of cancer cell lines from other origins, for example breast, colon, lung and neuroblastoma. In addition, in the last decade many studies have shown further effects that justify the expectation that triterpenes are useful to treat cancer by several modes of action. 

## 2. Ursane-Type Natural Triterpenes from Plants

Ursane type pentacyclic triterpenes abundantly exist in the plant kingdom. Of the ursane type pentacyclic triterpenes, ursolic acid (3β-hydroxy-urs-12-en-28-oic acid, **2**, Figure 2) is a prevalent pentacyclic triterpenoid. It has been found in various plants in both aglycone and glycoside forms, and traditional uses of plants containing **2** in folk medicine are abundant. Modern studies have shown that ursolic acid possesses many biological effects, such as anti-oxidative, anti-inflammatory, antitumor, and hepato-protective activity. The diverse inflammatory effects of ursolic acid were reviewed by Ikeda* et al.* in 2008 [13]. This review also summarized the inhibitory activity of ursolic acid on cancer cells. Ursolic acid proved to suppress the NF-κB pathway via inhibition of p65 phosphorylation, thereby causing down-regulation of the expression of downstream oncogenes. Compound ursolic acid may also reduce skin tumor formation by inhibiting the binding of carcinogen to epidermal DNA or cell membrane. Furthermore, ursolic acid induces cell differentiation and apoptosis in certain cancer cell lines. Ursolic acid exhibited chemopreventive effects during the cancer initiation phase of an* in vivo* inhibitory assay of aberrant crypt foci (ACF), which are putative precursors of colon cancer, and increased neutral sphingomyelinase activity [15]. Ursolic acid also inhibited endogenous reverse transcriptase (RT), an enzyme involved in the control of cell proliferation and differentiation, in melanoma (A375) and anaplastic carcinoma (ARO) cell lines. Down-regulation of the expression of two cancer-related genes, c-myc and cyclin-D1, in A375 and/or ARO cells was also stimulated by ursolic acid [16].

**Figure 3:**
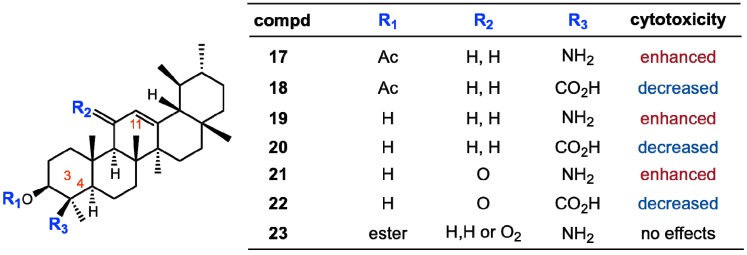
Substituent effects of boswellic acid analogs on cytotoxicity.

Several attempts were undertaken for the derivatization of ursolic acid seeking to obtain analogs with improved anti-tumor activity (Figure 2). Chao-Mei Ma* et al.* [17] modified the C-3, C-28, C-11 positions of ursolic acid (**2**). Among the 23 derivatives they synthesized, 3β-amino derivative **3** was found to be 20 times more potent than the parent ursolic acid on the HL-60, Bel-7402 and HeLa cell lines. Usually, compounds with β-oriented hydrogen-bond forming groups at C-3 exhibit more potent cytotoxicity than their α-counterparts. Besides, dimeric compounds **4** and **5** show selective cytotoxicity against HL-60 cell lines. Similarly, Shao* et al.* [18] also synthesized 23 derivatives by modifying at C-3 and the C-28 positions; significant improvement of the cell growth inhibition of human embryonic lung fibroblast cells (HELF) was achieved when an acetyl group was introduced at the 3-OH position, and also alkylamino and/or piperidine groups were introduced at the 17-COOH position in **6–9**. Their SAR studies also showed that a polar group at either the 3-OH and/or 17-COOH positions was essential for the cytotoxic activity. Alternatively, C-2 cyano or trifluoromethyl derivatives of 1-en-3-one-ursolic acid (compounds **10** and **11**) showed higher activity than C-2 iodo- and non-substituted analogues (compounds **12** and **13**) in antiproliferation assays using KU7, 253JB-V, Panc-1, and Panc-28 cancer cell lines (IC_50_: 0.17–1.13 mM) [19]. Among the natural ursane-triterpenes asiatic acid (**14**) was reported to significantly reduce the formation of skin tumors. Concurrently, **14** inhibited the tissue plasminogen activator (TPA), generation of NO, and expression of iNOS and COX-2, which are important factors in tumor promotion [20]. In addition, **14 **induced apoptosis in PPC-1 and U-87MG cancer cells. Two new ursane-type triterpenes, microfokienoxane C (**15**) and 3β,28-dihydroxy-11α-methoxyurs-12-ene (**16**) have recently been isolated from the leaves of *Microtropis fokienensis*; compound **15** showed cytotoxic activity against HepG2 and Hep3B cancer cell lines while compound **16** exhibited the activity against the HepG2 cell line [21]. Boswellic acids, containing the different type of pentacyclic core skeletons also exhibited the cytotoxic activities against several tumor cell lines of which the Structure Activity Relationship are summarized in Figure 3.

Thus, ursolic acid and its derivatives have been reported to show cytotoxicity against some cancer cell lines but have not been thoroughly explored in comparison to beutalinic acid derivatives and other lupanes. For examples, besides the derivatization of preexisting hydroxyl groups, their number and position in ursane triterpene structures, which may affect the cytotoxic activity, have not been investigated. Under such circumstances, recently Mazumder* et al.* [22] investigated the isolation of the biologically active compounds from the plant extracts of *Saurauja roxburghii.* This is an evergreen tree belonging to the family Dilleniaceae commonly found in Bangladesh, Butan, Northeast India, Nepal, Malaysia, Pakistan and Sri Lanka with various local names such as Sing krang, Sing khau,* etc.* Hereinafter Tanaka* et al.* [23] developed a ruthenium prophyrin-based catalytic oxidation procedure to obtain oxidized derivatives of ursolic acid, and additionally they investigated the cytotoxic activity against A431 and C6 cell lines both for the natural and semi-synthetic compounds. The following section reviews the isolation, structural elucidation, oxidative derivatization and evaluation of the cytotoxic activity of ursoilc acid and its derivatives.

### 2.1. Isolation of Triterpene Natural Products from Leaves of Saurauja roxburghii

**Figure 4:**
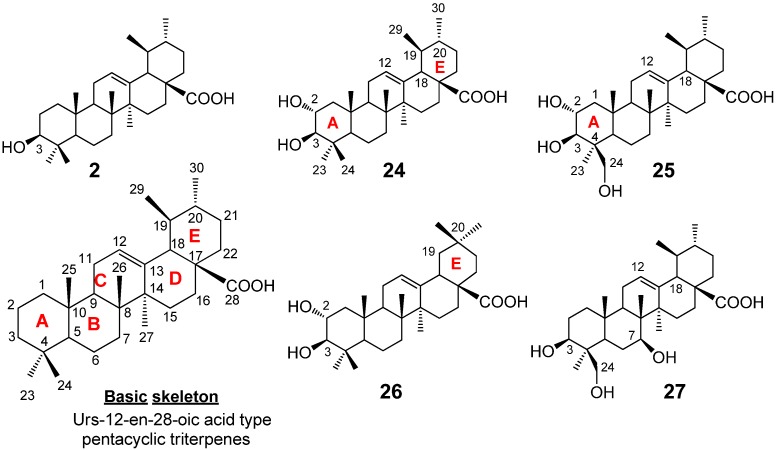
Ursolic acid derivatives isolated from *Saurauja roxburghii*.

Mazumder* et al.* [22] isolated pentacyclic triterpenes, mainly of the ursane type, from the sun-dried leaves of the plant *Saurauja roxburghii*, where powdered plant leaves were soaked in MeOH for seven days with occasional shaking and stirring (Figure 4). The whole mixture was then filtered and the filtrate was evaporated under the reduced pressure at 40–50 °C to give a gummy concentrate of the crude extract. The extract was subjected to solvent-solvent partitioning using conventional procedures. Then the chloroform fraction was subjected to the column chromatography on silica gel (sequentially eluted by *n*-hexane-chloroform (1:1), chloroform-methanol (9:1, 3:1, 1:3), and then methanol), and then subjected to the repeated reverse phase (RP)-HPLC, otherwise normal phase HPLC was used. Eventually five ursane-type triterpenes were isolated (Figure 4).

### 2.2. Structural Determination of Ursane-Type Triterpenes

As these urs-12-en-28-oic acids are widely available in the plant kingdom, their basic skeletons (see the basic structure in Figure 4) can easily be characterized by their common NMR spectroscopic features, e.g., the presence of: (1) the sharp singlet methyl protons at C-23, 24, 25, 26, and 27 and the two doublet upper field methyl protons at C-2 and C-3; (2) one olefinic proton signal for 12-H and a characteristic doublet proton signal for 18-H at around 2.5 ppm (*J* = 12 Hz), along with (3) the thirty carbon signals in ^13^C-NMR, which include two olefinic carbons for C-12 and C-13 and one down field carbonyl carbon for C-28 at around δ 180. Although Mazumder *et al.* [22] reported five terpenoids, of these four were previously known compounds. The authors pointed out that the simple comparison of their one-dimensional NMRs with the reported spectra was not sufficient to assign the structures, due to the similarity of all their structures, hence their ^1^H and/or ^13^C NMR signals, *i.e.*, only the differences in the number of hydroxyls and/or the position of the hydroxyl and the methyl substitutions in the same skeleton (see the structures in Figure 4) were distinguishable. Therefore, all two dimensional NMR experiments for each compound, such as ^1^H-^1^H COSY, HMQC, HMBC, and NOESY measurements were performed and compared with the reported data. Each structure was then determined as follows. Compound **2**, *i.e.*, ursolic acid, was obtained as a pale yellow amorphous solid. The ursane-12-en skeleton of **2** was established by the characteristic ^1^H-NMR signals of the two doublet methyl protons for 29-H and 30-H at 1.01 (3H, d,* J* = 6.0) and 0.96 (3H, d, *J* = 12.6), the 12-H vinyl proton at 5.49 (t) and the doublet methine proton (for 18-H) at 2.64 (d, *J* = 11.4 Hz). Stereochemistry of the 3-hydroxyl on the A-ring was elucidated by the observation of the doublet/doublet 3-H proton signal at 3.46 (*J* = 10.8, 5.4 Hz); such coupling constants indicated the 3-H proton was oriented as one axial/axial and one equatorial/axial orientations in the A-ring, hence the α-proton. This substitution was further confirmed by the ^13^C-NMR signal of C-3 at 78.2 ppm [24], and also supported by the ^1^H-^1^H COSY, HMQC, and HMBC experiments [25,26,27]. Compound **24**, corosolic acid, was obtained as a white amorphous solid. The ursane-12-en skeleton of this terpenoid was deduced as being similar to that of ursolic acid [28,29]. Specifically, the ^13^C-NMR analysis detected the two oxygenated methane signals at 69.1 (C-2) and 83.9 (C-3), which suggested a 2,3-dihydroxyl ursolic acid structure [30]; the positions and the relative configurations of these two hydroxyls, *i.e.*, the 2,3-dihydroxyls, was unambiguously concluded by 2D-NMR experiments such as ^1^H-^1^H COSY, HMQC and HMBC data. Compound **25**, 24-hydroxyl corosolic acid, was obtained as a white amorphous solid. This natural terpene contains the ursane-12-en skeleton with the two hydroxy substitutions at the C-2 and C-3, showing very similar NMRs to those of the corosolic acid **24**. The ^1^H-NMR showed the diastereotopic hydroxymethylene protons as two doublet protons at 3.37 and 4.01, which in turn exhibited the cross-peak correlation with a carbon signal at 66.2 in HSQC spectrum. The further analysis of NOE, HMBC and NOESY confirmed the structure as 2,3,24-trihydroxyl-urs-12-en-28-oic acid. Compound **26**, maslinic acid, was obtained as a white amorphous solid. The NMR data were very similar to those of the corosolic acid **24** except for the environment around the dimethyl groups in the E-ring; while two methyl signals in the corosolic acid **24** split into the doublets, they were both singlets in **26**. Additionally, no COSY correlations were observed among the 29-H, 19-H, 20-H, and 30-H as in **24**. Based on DEPT analysis, the compound **26** should contain the gem-dimethyl substituent at the C-20 position of the E-ring, hence leading to the maslinic acid structure, a positional isomer of corosolic acid. Compound **27** was reported as new compound, which was obtained as an amorphous solid. IR detected the representative absorption bands for the ursane-12-en structure, such as the hydroxyl (3,402 cm^−1^), carbonyl (1,684 cm^−1^), and olefinic (1,562 cm^−1^) groups. The ^1^H-NMR, ^13^C-NMR, and HMBC correlation suggested the structure of **27** being very similar to the 24-hydroxyl corosolic acid **24**. Thus, ^1^H-NMR detected the characteristic proton signals for the ursane-12-en skeleton, *i.e.*, one olefinic proton at 5.22, four singlet methyl proton at 0.81, 0.96, 1.08, and 1.13, and two doublet methyl protons at 0.88 (3H, d, *J* = 6.4) and at 0.96 (3H, d, *J* = 6.4).^13^C-NMR analysis also detected the two olefinic carbons at 126.7 and 139.7, in addition to the six methyl signals at 17.4, 17.6, 17.7, 21.6, 23.1, and 24.1. As the case of the other ursane-12-en-type structures **2**, **24**, **25**, the *cis*-fused D/E ring systems was also confirmed by the observation of the NOESY correlation between 12-H and 18-H. The observation of the carbon signals at 65.9 (methylene carbon based on DEPT analysis), 66.9 (methine carbon), and 74.6 (methine carbon), suggested the presence of three hydroxyls on the ursane-12-en skeleton; the oxymethine carbon at C-3 (74.6) was assigned through HMBC correlations with H- 23 (1.08) and H-24 (3.63). NOE between the 3-H and axial 1-H concluded the β-orientation of the hydroxyl at C-3. Alternatively, the oxymethylene at the β-substituted C-24 position (65.9) was assigned through the HMBC correlations with 23-H (1.08), 5-H (1.31), and 3-H (3.73), as well as the NOESY correlation with 1-H. The oxymethine at C-7 (66.9) was also assigned based on the HMBC correlations with 5-H (1.31) and 6-H (1.32); the NOESY correlation with axial 3-H and the bridgehead 27-H moreover determined the -axial orientation of 7-H (hence the β-hydroxyl at C-7). The^1^H-^1^H COSY and HMQC experiments were further carried out to assign all proton and carbon signals in **27**, giving rise to a new corosolic acid derivative, 3, 7, 24-trihydroxyl-urs-12-en-28-oic acid. This compound further been confirmed by ESI-QTOF MS/MS fragmentation analysis [31].

## 3. Biomimetic Oxidative Derivatization of Ursolic Acid

As described above, the hydroxylation patterns of ursane-type triterpenes significantly affects the cytotoxicity. These hydroxylated terpene metabolites are produced in many higher plants through the action of cytochrome P450 [32,33]. The resulting structural variants are responsible for the diverse range of the biological activities [34,35,36,37,38,39,40,41]. Therefore, chemical oxidation or hydroxylation of the terpenes may produce structural variants for further SAR studies. The authors envisioned that biomimetic oxidation using porphyrin derivatives, *i.e.*, the mimic of cytochrome P450, might diversify the core-structure of ursolic acid (**2**) [23]. The authors also pointed out that the auxiliary used on the oxidant structures affected the chemical and site selectivities,* i.e.*, the oxidation efficiency of the C–H, O–H, or olefins at the different positions of the target substrates. The following sections review the development of ‘Ru’-porphyrin catalysts, the oxidative derivatization of ursolic acid and modulation of the cytotoxicity towards different cancer cell lines.

### 3.1. Development of Auxillary-Directed ‘Ru’-tetraphenylporphyrin Derivatives and Oxidative Derivatization of Ursolic Acid

Selective oxygenation of saturated C-H bonds has been a major challenge in synthetic chemistry as exemplified by the pioneering work of Breslow and co-workers [42,43,44,45]. Lately, the regio- and stereo-selective oxidation of hydrocarbons at the late stage of the synthesis is the common trend in natural products synthesis [46]. However, it has still remained challenging to date due to the scarcity of the reagents that can efficiently and selectively oxidize the C-H bonds, such as those of the hydrophobic terpene skeleton. Under these circumstances, biomimetic oxidation using P450 variants and porphyrin derivatives offers an intriguing opportunity for diversifying the core-structure of terpenes [32,47,48,49]. In fact, the oxidation of ursolic acid derivatives by “Fe”-porphyrin has previously been examined by Konoike* et al.* [50].

**Scheme 1:**
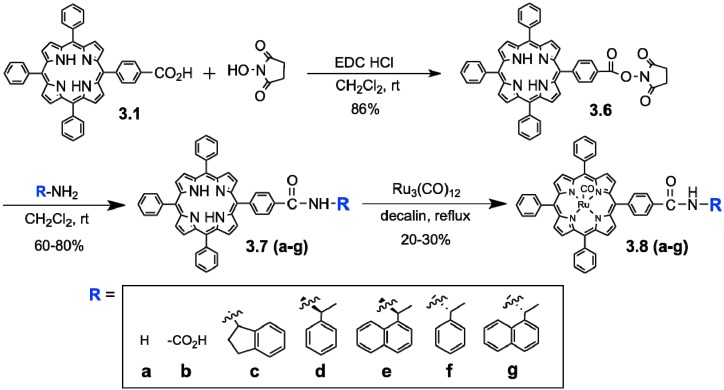
Synthesis of substituted “Ru”-porphyrin oxidants, Ru(TPP)(CO).

**Scheme 2:**
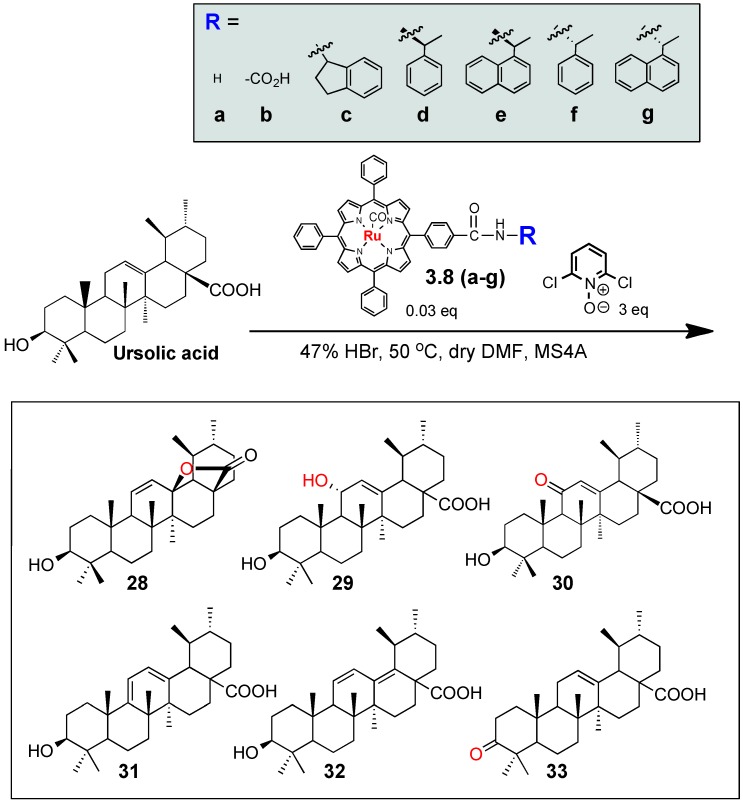
Oxidation of ursolic acid by “Ru”-porphyrin oxidants.

In the present study, we expected that the chemical and site-selectivities could be affected by the auxiliary on the oxidant structures,* i.e.*, oxidation efficiency of the C-H, O-H, or olefins at the different positions of the target substrates. We employed the ruthenium porphyrin derivatives **3.8a–g** as oxidation catalysts for the oxidation of ursolic acid **2** (Scheme 1). Previously, regio- and stereo-selective oxidation of steroids using “Ru”-porphyrin **3.8a** was reported by Nagano* et al.* [51], though “Ru”-porphyrin have been mainly used for epoxidation of alkenes [52]. “Ru”-porphyrins **3.8c–g**, which contain various chiral and non-chiral amides, were prepared from acid-functionalized tetraphenyl- porphyrin **3.8b** (Scheme 1). Ruthenium was incorporated into tetraphenylporphyrins **3.7a-g** by reacting with Ru_3_(CO)_12_ in hot decalin [53], providing **3.8a–g** in 20%–30% yields. Then, an *in situ* oxidation protocol was used employing porphyrins **3.8a–g** (Scheme 2) [23].

Thus, under the conditions given in Scheme 2, ursolic acid (**2**) was oxidized to afford variously oxidized products, including lactone **28** [54] C-11-hydroxy **29** [50,55] C-11-ketone **30** [56] dienes **31 **and** 32** [57] and C-3-ketone **33** [17]. All of the compounds **28–33** were known natural and/or chemically derivatized compounds. While the compound **33** was obtained by the oxidation of the C3-hydroxyl group of **2**, the compounds **28–32** could be derived by the allylic oxidation at C-11. We also obtained the small amounts of other oxidized products, which might be derived from the oxidation of the inactivated C-H bonds on the terpene skeleton, but enough amounts could not be obtained to determine their structures. 

We observed that the distribution of the oxidized products **28–33** was clearly affected by the auxiliaries of the ‘Ru’-porphyrins **3.8a–g**. Thus, the parent ‘Ru’-porphyrins **3.8a–g** gave the lactone **28** (25%) and the α-hydroxylated compound **29** (35%) as two major products (Table 1), but the production of **29** was significantly decreased by the acid derivative **3.8b** (4%), instead equally producing the other derivatives **30–32** (each about 10%); the introduction of the acid group on the metalloporphyrin might cause the interaction with the substrate **2**, for example, with the carboxylic acid or the hydroxyl groups in **2**, hence altering the oxidation selectivity. On the other hand, while the (*R*)-isomers **3.8d** and **3.8e** gave the α-hydroxylated **29** as the major product (entries 4 and 5), the reactivity of the corresponding (*S*)-oxidants **3.8f** and **3.8g** was significantly reduced (entries 6 and 7). For both cases, the oxidation efficiency was retarded by using the sterically more demanding 1-naphthyl derivatives **3.8e** and** 3.8g** (the starting compound **2** was recovered in 50% and 90% yield, respectively). Meanwhile, the ketone **33** was solely obtained when the indane derivatives **3.8c** was used (entry 3) [23]. This is the first observation that the auxiliaries on metalloporphyrin-based oxidants **3.8a–g** gave profound effects on the oxidation reactivity and selectivity, and even small functional groups on the side chains of the tetraphenylporphyrins, *i.e.*, acid, amides, or chirality, could recognize the triterpene structure **2**, and exhibited matched and/or mismatched combinations for the dioxoruthenium-catalyzed oxidation.

**Table 1:** Distribution of oxidized products by “Ru”-porphyrins.

Entry	Porphyrin catalyst	Product yields (%)						
		2 (recovery)	28	29	30	31	32	33
1	**3.8a**	25	25	35	5	ND	ND	ND
2	**3.8b**	35	35	4	8	10	8	ND
3	**3.8c**	80	ND	5	5	ND	ND	10
4	**3.8d**	ND	ND	70	5	ND	ND	ND
5	**3.8e**	50	ND	30	10	ND	ND	ND
6	**3.8f**	75	ND	5	5	ND	ND	ND
7	**3.8g**	90	ND	10	ND	ND	ND	ND

ND: not detected.

## 4. Evaluation of Cytotoxic Activity towards Tumor Cell Lines

We tested cytotoxicity of **2**, **24–27** against two tumor cell lines,* i.e.*, the human epidermoid carcinoma cell line, A431 and the rat glioma cell line, C6 [22]. The compounds **2**, **24–27** were dissolved in 100% ethanol and then diluted with Dulbecco's Modified Eagle's Medium (DMEM) as the working solutions which were added to the cells with a final volume of 0.2 mL (final concentration of 10–100 μM) and cultured over 0, 3 h, 6 h, 12 h, 24 h and 48 h, respectively [58]. The index of cell damage was adopted with the vacuolar degeneration and necrosis. We found that the ursolic acid (**2**) exhibited the cytotoxicity against C6 rat glioma at the concentrations of 10–100 μM, while this terpenoid did not show any activity towards the A431 carcinoma (Table 2). Meanwhile, corosolic acid (**24**) showed cytotoxicity against the both cell lines (Table 2). The cytotoxicity of corosolic acid to C6 glioma and A431 carcinoma had not been previously reported, though various biological activities of corosolic acid have been known [59]. Interestingly, the structural difference between ursolic acid (**2**) and corosolic acid (**24**) was the additional hydroxyl group at the C-2 in **24** (Figure 4). Namely, a single hydroxyl group on the A-ring of the ursane-12-en skeleton makes the cytotoxic activity selective against the C6 cell line. On the other hand, no cytotoxic activity could be observed for the other compounds **25–27** at the 10–100 μM concentration range (Table 2).

**Table 2:** Cytotoxicity of compounds **2**, **24–33** against two tumor cell lines, A431 human epidermoid carcinoma and C6 rat glioma. Cells were treated with 10 µM of the compound at 37 °C. “**+**” stands for positive and “−” stands for negative responses on morphological changes after 24 h.

Compound	A431 human epidermoid carcinoma	C6 rat glioma
**2**	−	+
**24**	+	+
**25**	−	−
**26**	−	−
**27**	−	−
**28**	+	+
**29**	−	−
**30**	−	−
**31**	−	−
**32**	−	−
**33**	−	−

Again, the simple modification of the cytotoxic **24** by introducing the hydroxyl group at C-24 (giving **25**) or by shifting the C-19 methyl to the C-20 (maslinic acid **26**), made the compounds inactive (Figure 4). These results clearly showed the importance of the position and numbers of hydroxyls or the methyl groups on the triterpene structure for the cytotoxic activity. It is further noted that the maslinic acid (**26**) is reported to show the cytotoxicity against HT29 human colon-cancer cells [31]; hence maslinic acid should exhibit selective activity to the HT29. Alternatively, asiatic acid (2α, 2β, 23-trihydroxy-urs-12-en-28-oic acid), the epimer of **25**, is a strong cytotoxic natural product against some cancel cells, such as the human glioblastoma cell lines U-87, human breast cancer cell lines MCF-7 (ATCC HTB-22), MDA-MD-231 (ATCC HTB-26), and the human hepatoma cell Hep G2 [24,60,61]. Thus, the tunable cytotoxicity and selectivity to the cell lines by the slight modification of their structures,* i.e.*, functional group substitutions and/or stereochemistry, make these triterpene natural products promising candidates for drug development and/or for tools for investigating the mode of action. On the other hand, for chemically oxidized compounds **28–33**, cytotoxicity was also examined as the same way as described for compounds **2**, **24–27**. It was observed that only the lactone **28** had cytotoxic activity against both the A431 and C6 tumor cell lines; therefore we can conclude that the oxidation at the C-11 and C-13 positions of ursolic acid (**2**), gave ursolic acid an additional cytotoxic activity against A431 tumor cell. Thus, the authors observed that the cytotoxic activity and the tumor selectivity of the natural and the chemically oxidized ursolic acids are sensitively modulated by simply modulating the oxidation states on the core terpene structure. 

## 5. Conclusions

Natural products are the most consistently successful sources of biologically diverse compounds, and especially, plants provide a large bank of rich, complex, and highly varied structures. Many higher plants contain densely oxidized terpene metabolites,* i.e.*, with carboxyl or hydroxyl groups, of which structural variants exhibit a diverse range of activities. Alternatively, the core terpene structures could also be derivatized by the chemical oxidation in pursuit of enhancing the activity and/or modulating the target selectivity,* i.e.*, against a specific tumor cell line. We investigated the isolation of the natural products from the extracts of the Bangladeshi medicinal plant*, Saurauja roxburghii,* a higher plant indigenous to south East Asia and some parts of North America. By using conventional extraction procedures, we isolated the five ursane-type pentacyclic triterpenes. The structures of these five compounds were unambiguously determined by extensive NMR and MS analyses, and also by comparisons with the literature data, if available. Then these natural triterpenes were tested for cytotoxicity against the C6 rat glioma and A431 human skin carcinoma cell lines. Very interestingly, despite the fact that these five compounds have the same ursane-type pentacyclic triterpene core structures, only ursolic acid (**2**) and corosolic acid (**24**) showed cytotoxicity at 10 μM concentrations. Furthermore, while the corosolic acid showed the cytotoxicity against both cell lines, ursolic acid exhibited selective cytotoxicity against the C6 glioma cell. These results clearly show that the position and numbers of hydroxyls on the terpene structure affect the activity and selectivity to the cancer cell lines. Inspired by these results, our group sought to diversify the hydrophobic ursane-type triterpene ursolic acid, for further SAR studies, by applying chemical oxidation using “Ru”-porphyrins. The newly developed auxiliary catalyzed ‘Ru’-tetraphenylporphyrins chemical oxidation system could produce oxidized ursolic acid derivatives could give a new direction in designing the porphyrin-based reagents as well as other oxidants. It is noted that the chemically oxidized products **28–33** were all naturally occurring compounds, but were difficult to isolate from the *S. roxburghii* in previous investigations, thus the chemical protocol can be complementary to the isolation of natural products.
